# Palmitic and Stearic Acids Inhibit Chaperone-Mediated Autophagy (CMA) in POMC-like Neurons In Vitro

**DOI:** 10.3390/cells11060920

**Published:** 2022-03-08

**Authors:** Rodrigo Espinosa, Karla Gutiérrez, Javiera Rios, Fernando Ormeño, Liliana Yantén, Pablo Galaz-Davison, César A. Ramírez-Sarmiento, Valentina Parra, Amelina Albornoz, Iván E. Alfaro, Patricia V. Burgos, Eugenia Morselli, Alfredo Criollo, Mauricio Budini

**Affiliations:** 1 Molecular and Cellular Pathology Laboratory, Institute in Dentistry Sciences, Dentistry Faculty, University of Chile, Santiago 8380544, Chile; rodrigo.espinosa.lemunao@gmail.com (R.E.); karla.gutierrezo@utem.cl (K.G.); javierariosc@gmail.com (J.R.); fernandoou@ug.uchile.cl (F.O.); 2 Centro Ciencia & Vida, Fundación Ciencia & Vida, Avda. Zañartu 1482, Ñuñoa, Santiago 7780272, Chile; lilianayanten@gmail.com (L.Y.); aalbornoz@cienciavida.org (A.A.); ialfaro@udd.cl (I.E.A.); patricia.burgos@uss.cl (P.V.B.); 3 Institute for Biological and Medical Engineering, Schools of Engineering, Medicine and Biological Sciences, Pontificia Universidad Católica de Chile, Santiago 7820436, Chile; pagalaz@uc.cl (P.G.-D.); cesar.ramirez@uc.cl (C.A.R.-S.); 4 Advanced Center for Chronic Diseases (ACCDiS), Facultad de Ciencias Químicas y Farmacéuticas & Facultad de Medicina, Universidad de Chile, Santiago 8380544, Chile; vparra@ciq.uchile.cl (V.P.); alcriollo@u.uchile.cl (A.C.); 5 Autophagy Research Center (ARC), Santiago 8380544, Chile; emorselli@bio.puc.cl; 6 Departamento de Ciencias Básicas, Facultad de Medicina y Ciencia, Universidad San Sebastián, Lota 2465, Santiago 7510157, Chile; 7 Programa de Comunicación Celular en Cáncer, Instituto de Ciencias e Innovación en Medicina, Facultad de Medicina, Clínica Alemana Universidad del Desarrollo, Santiago 7610658, Chile; 8 Centro de Biología Celular y Biomedicina (CEBICEM), Facultad de Medicina y Ciencia, Universidad San Sebastián, Lota 2465, Santiago 7510157, Chile; 9 Centro de Envejecimiento y Regeneración (CARE-UC), Facultad de Ciencias Biológicas, Pontificia Universidad Católica, Santiago 8331150, Chile; 10 Laboratory of Autophagy and Metabolism, Department of Physiology, Faculty of Biological Sciences, Pontificia Universidad Católica De Chile, Santiago 8331150, Chile; 11 Cellular and Molecular Biology Laboratory, Institute in Dentistry Sciences, Dentistry Faculty, University of Chile, Santiago 8380544, Chile

**Keywords:** chaperone-mediated autophagy (CMA), insulin, palmitic, stearic, lysosome, obesity, proteomics, SILAC

## Abstract

The intake of food with high levels of saturated fatty acids (SatFAs) is associated with the development of obesity and insulin resistance. SatFAs, such as palmitic (PA) and stearic (SA) acids, have been shown to accumulate in the hypothalamus, causing several pathological consequences. Autophagy is a lysosomal-degrading pathway that can be divided into macroautophagy, microautophagy, and chaperone-mediated autophagy (CMA). Previous studies showed that PA impairs macroautophagy function and insulin response in hypothalamic proopiomelanocortin (POMC) neurons. Here, we show in vitro that the exposure of POMC neurons to PA or SA also inhibits CMA, possibly by decreasing the total and lysosomal LAMP2A protein levels. Proteomics of lysosomes from PA- and SA-treated cells showed that the inhibition of CMA could impact vesicle formation and trafficking, mitochondrial components, and insulin response, among others. Finally, we show that CMA activity is important for regulating the insulin response in POMC hypothalamic neurons. These in vitro results demonstrate that CMA is inhibited by PA and SA in POMC-like neurons, giving an overview of the CMA-dependent cellular pathways that could be affected by such inhibition and opening a door for in vivo studies of CMA in the context of the hypothalamus and obesity.

## 1. Introduction

It is well known that the consumption of high-fat diets (HFDs) containing high levels of saturated fatty acids (SatFAs) can lead to obesity and related disorders, such as insulin resistance [1,2]. A population of hypothalamic neurons is in charge of regulating the food intake balance by sensing circulating lipids and glucose, but changes in this process may lead to metabolic disorders and obesity [3]. Previous studies have shown that SatFAs, especially palmitic acid (PA), accumulate in the hypothalamus, causing inflammation, depression, and neuronal stress [4,5,6]. 

Autophagy is a lysosomal-dependent degradation pathway involved in the recycling of functional and unfunctional proteins and organelles [7]. Every cell has a basal autophagy activity that can be up- or downregulated according to the physiological state of the cell. Several studies have indicated that autophagy must be finely regulated as an imbalance in its activity, either positively or negatively, has been associated with diseases, such as cancer [8], neurodegeneration [9], and cardiovascular and metabolic disorders [10,11]. Autophagy can be divided into macroautophagy (most known as autophagy), microautophagy, and chaperone-mediated autophagy (CMA) [7]. CMA degrades proteins but not organelles and does not require the formation of intermediate vesicles (like macroautophagy) [12,13]. Importantly, proteins that are degraded by CMA must contain at least one motif related to the pentapeptide KFERQ (KFERQ-like motif), which is recognized by the HSC70 chaperone for its lysosomal degradation [12,14,15,16]. The HSC70-target protein complex interacts with the lysosomal transmembrane protein LAMP2A, which finally internalizes the protein to be degraded in the lysosomal lumen [14,15,17]. 

HFD has been observed to alter the activity of macroautophagy in tissues, such as the kidney [18], heart [19], and hypothalamus [20,21]. At the mechanistic level, studies have shown that proopiomelanocortin hypothalamic neurons (POMC) treated with PA impair autophagosome and lysosome fusion through activation of the free fatty acid receptor 1 (FFAR1) [22,23]. In addition, macroautophagy has been shown to regulate insulin signaling at the hypothalamic levels. Experiments in obese mice showed both a reduction in macroautophagy-related genes (atg 5 and atg 7), and the in vivo downregulation of these genes (particularly atg 7) lead to insulin resistance [24] and a reduction of insulin secretion in β cells [25]. Further, the in vitro treatment of POMC neurons with PA affected the insulin response as a consequence of macroautophagy disfunction [23]. 

The relationship between CMA and obesity has been less studied. In this respect, HFD consumption decreases CMA activity in mice liver by destabilizing LAMP2A complexes at the lysosomal membrane and by accelerating the degradation of this protein [26]. In the hypothalamus, changes in the expression of LAMP2A and HSC70 occur in mice after HFD consumption, and in vitro through the treatment of hypothalamic cells with palmitate [27]. However, it remains unclear whether CMA activity is altered under obesogenic conditions in the hypothalamic context and, further, the consequences of such CMA activity alteration. 

In this work, we used a POMC-like cell line (N43/5) to study the effect of SatFAs, such as palmitic and stearic acids (PAs and SAs, respectively), on CMA activity. Further, from a proteomic-based analysis of isolated lysosomes, we investigated the putative CMA-dependent pathways that could be affected by PA and SA exposure. We found that PA and SA inhibit CMA activity, probably by reducing the total and lysosomal LAMP2A protein levels. In addition, we observed that the principal pathways that could be affected by dysregulation of CMA, under PA and SA exposure, correspond to mitochondria-related processes (translation, β-oxidation), vesicle formation and trafficking, endoplasmic reticulum, and Golgi crosstalk, among others. Importantly, we show for the first time that CMA activity inhibition affects the insulin response of POMC neurons. Overall, these results confirm that an obesogenic condition with SatFAs excess can inhibit CMA activity in POMC neurons in vitro and consequently affect important cellular processes. This work contributes to our understanding so that CMA activity is considered in studies aiming to dissect the pathological consequences of HFD and SatFAs on hypothalamic function.

## 2. Material and Methods

### 2.1. Cell Culture

Embryonic Mouse Hypothalamic N43/5 cell lines were grown in DMEM (Hyclone SH30243.02 Cyvita, Marlborough, MA, USA) supplemented with 10% fetal bovine serum (GIBCO, Thermo Fisher Scientific, Waltham, MA, USA) and antibiotic-antimycotic solution (GIBCO 15240-062, Thermo Fisher Scientific, Waltham, MA, USA) at 37 °C in a humidified environment with 5% CO_2_. Serum deprivation (0% FBS) consisted in washing the cells carefully 2 or 3 times with PBS and cultivating them in DMEM 0% FBS plus antibiotic-antimycotic for the specified hours. 

### 2.2. LAMP2A and HSC70 Protein Levels after PA and SA Treatment

N43/5 (2.5 × 10^5^) cells were cultured in 6-well plates. The day after, the culture medium was changed to DMEM 2% FBS for 16 h and 100 μM PA or SA (or the same volume of BSA) was added for 2, 4, or 6 h. After the fatty acids incubation time, the cells were collected, lysed in RIPA buffer, and the LAMP2A and HSC70 protein levels were evaluated by Western blot using LAMP2A (Abcam #ab18528, Cambridge, UK,), HSC70, (Invitrogen #13D3, Thermo Fisher Scientific, Waltham, MA, USA), and β-ACTIN (Sigma-Aldrich #A3854, Merck Bulgaria EAD, Sofia, Bulgaria) antibodies.

### 2.3. RT-qPCR

The protocol described in Section 2.2 was performed and total RNA was extracted by Trizol (Invitrogen, Thermo Fisher Scientific, Waltham, MA, USA) according to the manufacturer’s instructions. RNA was treated with DNase I (Invitrogen, Thermo Fisher Scientific, Waltham, MA, USA) and then quantified. Reverse transcription was performed using 1 mg of total RNA and random primers (Thermo Scientific, Thermo Fisher Scientific, Waltham, MA, USA) in 20 μL of reaction mixture using the reverse transcription reagents kit (Invitrogen, Thermo Fisher Scientific, Waltham, MA, USA). For PCR, a KAPA SYBR FAST qPCR (KAPABIOSYSTEMS, Sigma-Aldrich, Merck Bulgaria EAD, Bulgaria) kit was used. Reactions were made in triplicate using 2 μL of cDNA and a 0.4 μM final concentration of each primer in a 25 μL final volume. The following primers were used: *β*-*actin*, sense (5′-GATCTGGCACCACACCTTCT-3′) and antisense (5′-GGGGTGTTGAAGGTCTCAAA-3′); m*lamp2a*, sense (5′-AGGTGCTTTCTGTGTCTAGACCGT-3′) and antisense (5′-AGA ATA AGT ACT CCT CCC AGA GCT GC-3′). Reactions were subjected to dissociation curve analysis to exclude the possibility of nonspecific amplification. Changes for each gene were calculated using the mean of the change in the Ct values (ΔCt) normalized to the Ct values of *β-actin* for each sample (2^−ΔΔCt^).

### 2.4. GAPDH Protein Level Evaluation after PA and SA Treatment

N43/5 cells (3 × 10^5^) were cultured in 6-well plates and the day after, the cells were subjected to serum deprivation for 16 or 20 h. The cells were collected, lysed in RIPA buffer, and the GAPDH protein levels were evaluated by Western blot using GAPDH (Santa Cruz #sc-365062, CA, USA) and β-ACTIN antibodies.

N43/5 (2.5 × 10^5^) cells were plated in 6-well plates. The day after, the culture medium was changed to DMEM 2% FBS for 16 h and 100 μM PA or SA (or the same volume of BSA) was added for 6 h. Next, the cells were subjected to serum deprivation for 16 h. Finally, the cells were collected, lysed in RIPA buffer, and the levels of GAPDH evaluated by Western blot as described before.

### 2.5. PA-mCherry-KFERQ Immunofluorescence Assay

For the experiments, cells were seeded in optical 96-well plates and cultured with DMEM 2% FBS serum before the transfection. Cells were transfected with 0.5 µg of PA-mCherry-KFERQ plasmid (created by Dr. Iván Alfaro, Fundación Ciencia & Vida, Santiago, Chile) DNA using Lipofectamine 2000 (Invitrogen, Thermo Fisher Scientific, Waltham, MA, USA) for 4 h at 1.2 µL per µg of DNA. After 48 h post-transfection, the cells were treated with 100 µM PA or SA (or the same volume of BSA) for 6 h and then starved overnight. 

The cells were photoactivated and then fixed with 4% PFA solution plus 4% sucrose for 20 min at room temperature, washed 3 times with PBS Ca^2+^/Mg^2+^, and nuclei were stained with DAPI. Fluorescence microscopy images were acquired using an Olympus FV1200 confocal microscope using a 60× AN 1.4 water immersion objective at a 2.048 × 2.048-pixel resolution. For image analysis, a semiautomatic single cell and particle segmentation method was programmed in ImageJ Fiji software to quantify the number of puncta per cell. These measurements incorporate a watershed algorithm for segmentation of the cell cytoplasm and a local thresholding method using the Phansalkar algorithm for PA-mCherry-KFERQ-positive particle analysis.

### 2.6. Lysosomal and Mitochondrial Fractionation and SILAC Analysis

The protocol for lysosomal isolation was reported in Ormeño et al. 2020 [28]. Briefly, cells were cultured in 150-mm plates and after washing with cold PBS collected by centrifugation at 500× *g* for 5 min at 4 °C. Then, cells were resuspended in 2 mL of 2.5 M sucrose pH 7.2 and lysed by nitrogen cavitation. Samples were centrifuged at 2500× *g* for 15 min at 4 °C and postnuclear supernatant (PN) placed on the top of a Nycodenz gradient (2 mL 35% Nycodenz/0.25 M Sucrose, 2 mL 17% Nycodenz/0.25 M Sucrose, 3 mL Percoll/0.25 M Sucrose) and spun down at 20,000 rpm for 35 min at 4 °C. Lysosomal/mitochondrial interphase was collected and placed in a new ultra-clear tube and mixed with 80% Nycodenz to achieve a density of 35%. A new gradient of Nycodenz was generated (2 mL 35% Lysosomal/mitochondrial sample, 2 mL 17% Nycodenz/0.25 M Sucrose, 2 mL 5% Nycodenz/0.25 M Sucrose) by ultracentrifugation, as described above. Mitochondria and lysosomal fractions were separated (first and second band from the bottom, respectively) and collected from the interphases generated. Samples were washed with 1 volume of 0.25 M sucrose and centrifuged at the maximum velocity (4500× *g*) for 15 min at 4 °C. The supernatant was discarded and pellets (10–20 μg) were used to evaluate the fractionation by Western blot using the antibodies LAMP2A (Abcam #ab18528, Cambridge, UK), LAMP1 (Cell Signaling #9091S, Beverly, MA, USA), CATD (R&D Systems #AF1014, Minneapolis, MN, USA), VDAC (Cell Signaling #4866S. Beverly, MA, USA), and MTCO1 (Abcam #ab1852890668, Cambridge, UK).

According to the manufacturer’s protocol, for the SILAC analysis, the cells treated with PA and SA were incubated with Heavy DMEM/FBS mixture (SILAC Lys6 Arg6 KIT 282926433 from SILANTES, Munich, Germany). The control cells (BSA) were incubated with cold amino acids. Once the lysosomal purification was performed (as described before), 25 μg of lysosomal control sample BSA were combined with 25 μg of PA or SA lysosomal sample (50 μg total). The PA and SA samples (50 μg each one) were analyzed by mass spectrometry. 

### 2.7. Mass Spectrometry and Data Analysis

Mass spectrometry was performed by the Plateforme Protéomique Structurale et Fonctionnelle, Institut Jacques Monod—CNRS et Université Paris-Diderot (France). The lysosomal samples (50 μg of PA and 50 μg of SA) were resuspended in RIPA buffer. The samples were precipitated, digested with trypsin overnight, and run in 3 technical triplicates. Data analysis was performed by Mascot 2.5.1 coupled to Proteome Discoverer 2.2 and the Swissport database. Then, the obtained data were filtered using the following parameters: (i) proteins identified with high false discovery rates (FDRs) and with more than 1 peptide, (ii) proteins with a *p*-value less than 0.05, and (iii) proteins with a fold change of 2 (Supplementary Table S1).

### 2.8. KFERQ-like Motifs Containing Proteins and STRING Analysis

The data in Supplementary Table S2 were analyzed via the website https://rshine.einsteinmed.org (accessed on 9 February 2022) to identify proteins containing KFERQ-like motifs. The data were collected and graphed according to (i) the total proteins containing KFERQ-like motifs in the PA and SA treatments, (ii) the up- and downregulated proteins containing KFERQ-like motifs in the PA and SA treatments, and (iii) the proteins with different KFEQR-like motifs in the PA and SA treatments (Figure 5 and Supplementary Table S2). Proteins identified as “other kinds of motifs” (Supplementary Table S2) were considered as no-motifs.

The up- and downregulated proteins in PA and SA containing KFERQ-like motifs (Supplementary Table S2) were analyzed by the STRING website (https://string-db.org accessed on 9 February 2022) to identify the cellular pathways in which they interacted and participated in. Three interacting clusters were chosen in STRING (shown in red, green, and blue in Figure 6) and the words representing the main pathways of each cluster of the proteins were indicated.

### 2.9. Preparation of the N45/3 Cell Line with Reduced Levels of LAMP2A Protein

Lentiviral particles were generated by calcium phosphate transfection of HEK293-T cells with the plasmids pCMV-dR8.91, VSV-G, and pSUPER-shlamp2a [29] (kindly gifted by Dr. Ana María Cuervo, Albert Einstein College of Medicine, Bronx, NY, USA). N43/5 cells were infected with the lentiviral particles and different cells expressing the GFP protein were selected for further amplification and LAMP2A protein level evaluation through Western blot using anti-LAMP2A and β-ACTIN antibodies. 

### 2.10. Insulin Treatment

The insulin treatment was performed as previously described by Hernández-Cáceres et al. 2019 [23]. Specifically, 1 × 10^5^ N43/5 cells were plated in DMEM 10% FBS and 24 h later, the culture medium was changed to DMEM 0% FBS. After overnight incubation, the cells were treated with 100 μM of PA or SA (or the same volume of BSA) for 6 h. Next, the cells were washed with PBS and incubated for 13–15 min with DMEM 0% FBS supplemented with 1 μM of insulin (Humulin R). The insulin stimulus was stopped by incubating the cell plate on ice, immediately washing the cells with PBS, and adding the buffer lysis (RIPA). The insulin stimulus was detected by Western blot using 30–40 μg of total cell lysate and the antibodies against phosphorylated AKT (p-Ser473 Cell Signaling #9271 Beverly, MA, USA), total AKT (Cell Signaling #9272, Beverly, MA, USA), and β-ACTIN. 

### 2.11. Statistics

Data analysis was performed from at least three independent experiments, obtained separately from different sets of cell cultures. Different statistical methods were applied. When two groups were analyzed, we used an unpaired nonparametric *t*-test or ordinary one-way ANOVA. Numerical results were reported as ± SEM and the *p*-values corresponded to *p* < 0.05. When more than two groups were compared, mixed-effect analysis or two-way ANOVA were used. Numerical results were reported as ± SEM and the *p*-valued corresponded to *p* < 0.05. Prism 9, (GraphPad Software, San Diego, CA, USA) was used to perform the statistics analysis. For Western blot densitometric analysis, ImageJ software was used.

## 3. Results

### 3.1. Total and Lysosomal LAMP2A Protein Levels Decrease in POMC-like Neurons Treated with Palmitic and Stearic Acids 

**Figure 1:**
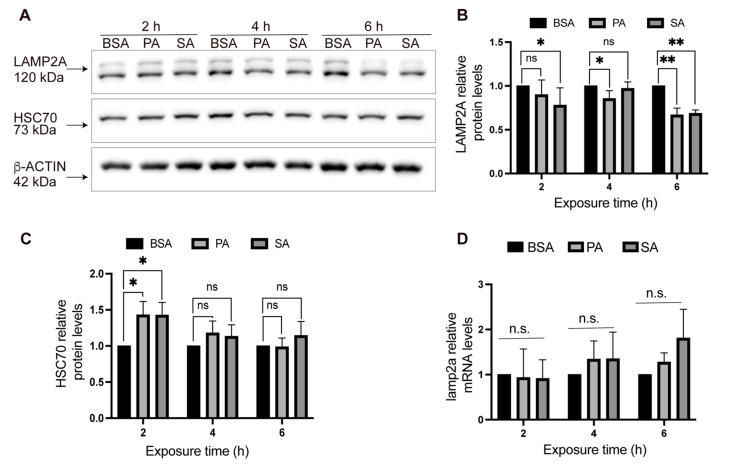
Total LAMP2A protein levels decrease in POMC-like neurons treated with PA and SA. (**A**) POMC-like neurons (N43/5) were incubated with 100 mM of PA, SA, or vehicle (BSA) for 2, 4, and 6 h and the total protein levels of LAMP2A, HSC70, and β-ACTIN were evaluated by Western blot. (**B**) Quantification of LAMP2A from Figure 1A where each time point was analyzed independently (e.g., BSA vs. PA at 2 h) using the Kolmogorov–Smirnov nonparametric *t*-test (*n* = 4). (**C**) Quantification of HSC70 from Figure 1A where each time point was analyzed independently (e.g., BSA vs. PA at 2 h) using the Kolmogorov–Smirnov nonparametric *t*-test (*n* = 4). (**D**) POMC-like neurons (N43/5) were incubated with 100 mM of PA, SA, or vehicle (BSA) for 2, 4, and 6 h and the total mRNA levels of *lamp2a* were evaluated by RT-qPCR. The data were analyzed by mixed-effect analysis (*n* ≥ 3). n.s. = non-significant, * = *p* < 0.05, ** = *p* < 0.01.

Previous results have shown that palmitic acid inhibits macroautophagy in hypothalamic POMC-like cells [22,23]. Regarding CMA, a recent work showed a decrease in the LAMP2A staining intensity after palmitate treatment in hypothalamic neurons [27]. However, the effects of SatFAs on CMA activity in hypothalamic neurons remain unstudied. To explore this question, we treated hypothalamic POMC-like cells with BSA (serum bovine albumin) and 100 μM of PA and SA at different time points (0, 2, 4, and 6 h) and evaluated the total protein levels of the principal CMA players (LAMP2A and HSC70) by Western blot post SatFAs treatment. We chose 100 μM of PA and SA because this is the concentration of PA that has been observed to accumulate in the hypothalamus of mice fed an HFD [30] and because this is the concentration reported to inhibit macroautophagy in POMC-like neurons [22,23]. Compared with BSA, we observed that the total protein levels of LAMP2A, but not HSC70, decreased after 6 h of PA and SA treatment (Figure 1A–C). Under the same conditions, we also evaluated the *lamp2a* mRNA levels; however, compared with BSA, we did not observe significative changes in this parameter (Figure 1D). 

**Figure 2:**
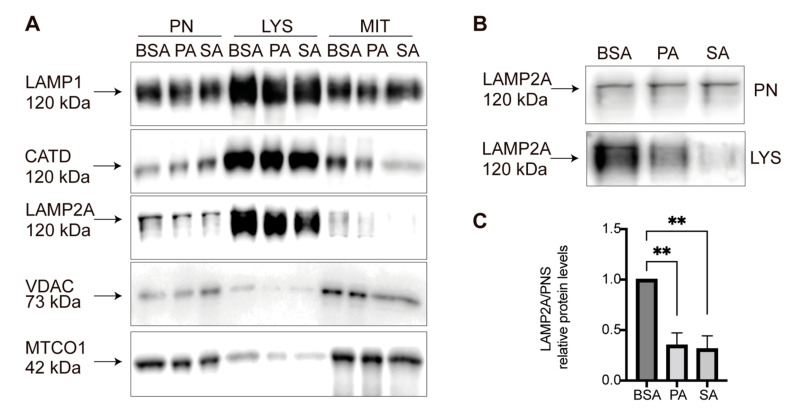
Lysosomal LAMP2A protein levels decrease in POMC-like neurons treated with PA and SA. (**A**) POMC-like neurons (N43/5) were incubated with 100 mM of PA, SA, or vehicle (BSA) for 6 h and a mitochondrial and lysosomal fractionation was carried out according to Ormeño et al. 2020. Post nuclear (PN), lysosomal (LYS), and mitochondrial (MIT) fractions were evaluated by Western blot using LAMP1, CATD, LAMP2, VDAC, and MTCO1 antibodies, as appropriate. (**B**) Representative Western blot comparing the lysosomal LAMP2A protein levels versus the PN fractions after BSA, PA, and SA treatment. (**C**) Quantification of LAMP2A from Figure 2B using ordinary one-way ANOVA (*n* = 3). n.s. = non-significant, ** = *p* < 0.01.

The CMA activity status depends on the abundance of LAMP2A, principally at the lysosomal membrane [26,31,32]. Thus, we asked whether LAMP2A protein levels changed in the lysosomes of POMC-like neurons treated with PA and SA. We purified lysosomes from POMC-like cells treated with BSA or 100 μM of PA and SA for 6 h to obtain different fractions enriched for mitochondria (VDAC and MTOC1) and lysosomes (CATD, LAMP1, and LAMP2A) (Figure 2A). Compared with BSA, a decrease in LAMP2A protein levels was observed in the lysosomal fractions treated with PA and SA (Figure 2B,C). 

Altogether, these results indicate that PA and SA treatment reduces the total and lysosomal LAMP2A protein levels in POMC-like neurons. 

### 3.2. Chaperone-Mediated Autophagy (CMA) Activity Is Inhibited in POMC Neurons Exposed to Palmitic and Stearic Acids 

**Figure 3:**
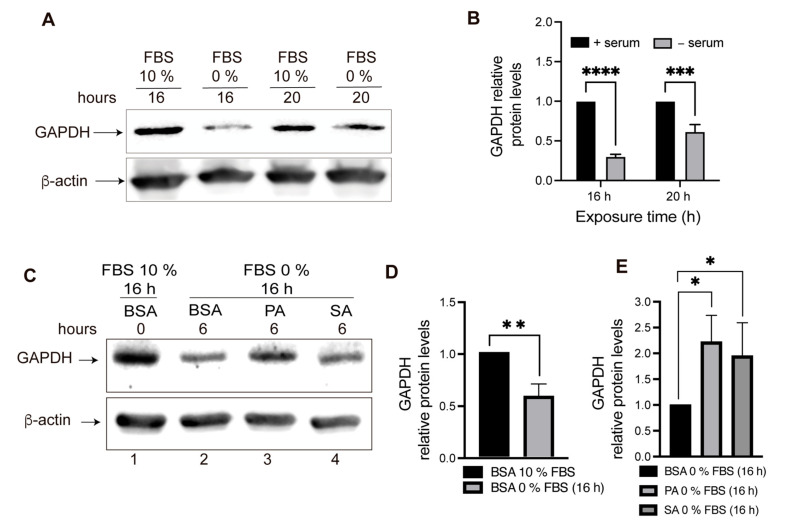
GAPDH protein levels, a well-known CMA substrate, increase in POMC-like neurons treated with PA and SA. (**A**) POMC-like neurons (N43/5) were serum deprived (0% FBS) or not (10% FBS) for 16 or 20 h. Then, total GAPDH protein levels were evaluated by Western blot as an indication of the CMA activity status. β-ACTIN was used a loading control. (**B**) Quantification from Figure 3A using two-way ANOVA (*n* = 5). (**C**) POMC-like neurons (N43/5) were incubated with 100 mM of PA, SA, or vehicle (BSA) for 6 h and then serum deprivation (0% FBS) was carried out for 16 h to induce CMA (as a control, cells were incubated for 16 h with 10% FBS). Then, the total GAPDH protein levels were evaluated by Western blot as an indication of the CMA activity status. β-ACTIN was used as a loading control. (**D**) Quantification of lines 1 and 2 from Figure 3C using the Kolmogorov–Smirnov nonparametric *t*-test (*n* = 6). (**E**) Quantification of lines 2, 3, and 4 from Figure 3C using the Kolmogorov–Smirnov nonparametric *t*-test (*n* = 6). n.s. = non-significant, * = *p* < 0.05, ** = *p* < 0.01, *** = *p* < 0.001, **** = *p* < 0.0001.

It is well established that LAMP2A protein levels, particularly in lysosomes, are the limiting step of CMA activity [26,31,32]. Thus, we asked whether the observed reduction in total and lysosomal LAMP2A protein levels correlated with a decrease in CMA activity. To characterize the response of CMA activity, we first evaluated the protein levels of GAPDH, a well-known CMA substrate [31,32]. We stimulated the CMA activity by serum deprivation (0% FBS) for 16 and 20 h. We observed a clear decrease in the GAPDH protein level at 16 h of serum deprivation, indicating that POMC-like cells activate CMA under these conditions (Figure 3A,B). Next, to evaluate the effect of PA and SA on CMA activity, we treated the cells with 100 μM of these SatFAs for 6 h and then stimulated the CMA activity by 16 h of serum deprivation. Whereas the serum deprivation reduced the GAPDH protein levels in the presence of BSA, the levels of GAPDH remained almost unaltered in cells treated with PA and SA (Figure 3C,D), suggesting an inhibition of CMA activity. 

**Figure 4:**
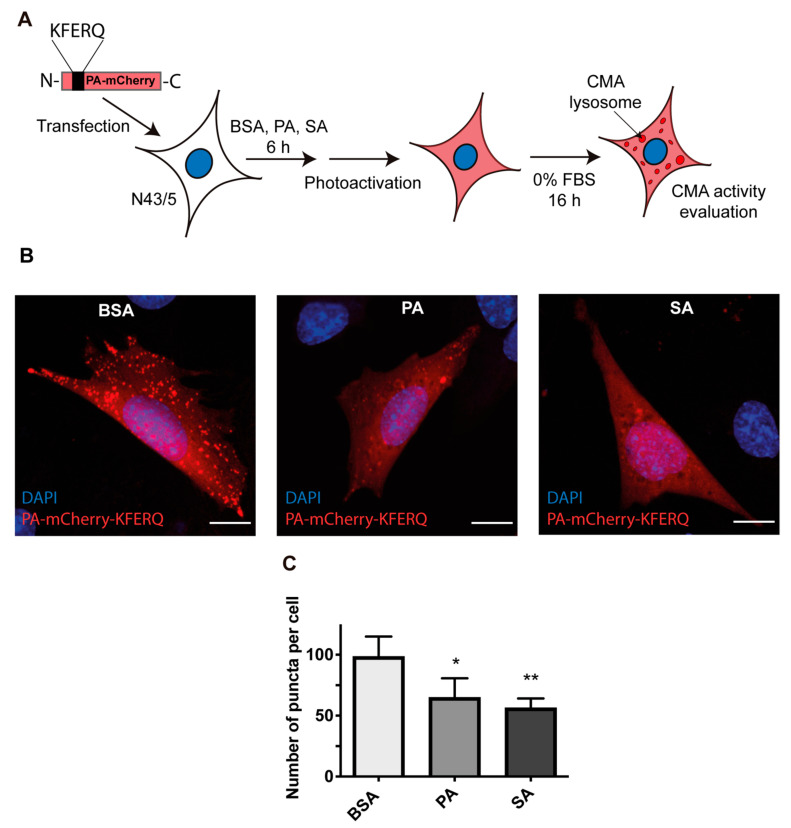
CMA activity, measured by a specific CMA fluorescent reporter, is inhibited in POMC-like neurons treated with PA and SA. (**A**) Cells were transfected with PA-mCherry-KFERQ plasmid DNA, treated with BSA or 100 µM of PA or SA for 6 h, and then photoactivated and starved for 16 h. (**B**) Representative fluorescent images showing the PA-mCherry-KFERQ puncta (red) in BSA, PA, or SA conditions (blue corresponds to cell nuclei). Scale bar = 20 μm. (**C**) Puncta per cells were quantified by ImageJ 1.8.0 (National Institutes of Health, NIH, MD, USA) and analyzed using the unpaired *t* test (*n* = 4) * = *p* < 0.05, ** = *p* < 0.01.

To confirm the inhibition of CMA activity by PA and SA, we used a specific fluorescent CMA reporter previously described [33]. This reporter consists of a photoactivable mCherry fluorescent protein fused to a KFERQ classic motif (PA-mCherry-KFERQ). This reporter is visualized as red foci in the cells (each focus indicates an active CMA lysosome) and the number of red foci in the cell is a readout of the CMA activity status (the more red foci→ more CMA activity) [33]. To measure the CMA activity through the PA-mCherry-KFERQ reporter, the cells were incubated with BSA, PA, or SA (100 μM) for 6 h; the reporter was photoactivated; and then the cells were subjected to serum deprivation for 16 h to quantify the number of red foci (Figure 4A). Upon serum deprivation, and compared with BSA, the cells treated with PA and SA showed a decrease in the number of PA-mCherry-KFERQ foci (Figure 4B,C). Altogether, these data indicate that, under the conditions of this study, CMA activity decreases in POMC-like cells treated with PA and SA. 

### 3.3. Putative CMA Protein Substrates in Lysosomes Isolated from POMC-like Cells Treated with PA and SA

**Figure 5:**
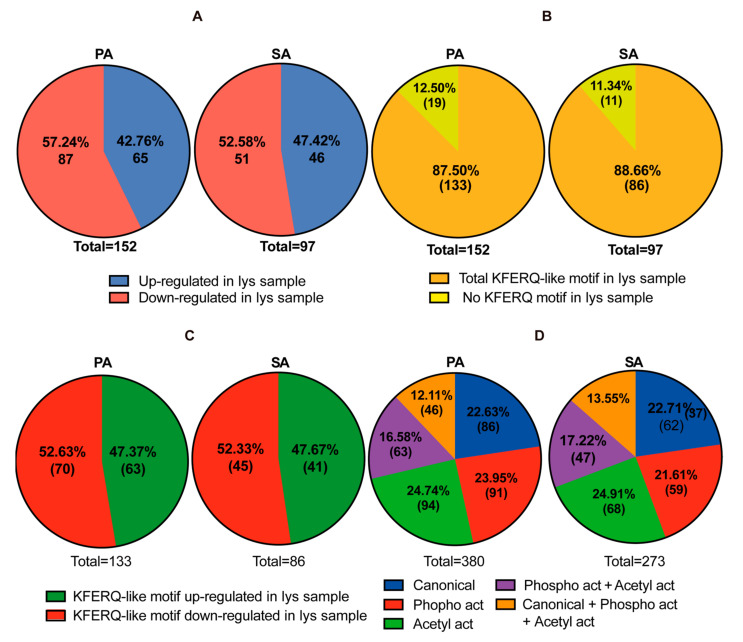
Analysis of putative CMA protein substrates in lysosomes from POMC-like neurons treated with PA and SA. POMC-like neurons (N43/5) were incubated with heavy DMEM/FBS mixture (SILAC Lys6 Arg6) and then incubated with 100 mM of PA, SA, or vehicle (BSA) for 6 h. Lysosomal fractionations were isolated from each condition as described in Figure 2 and their proteomic composition was analyzed by SILAC-based proteomics. The data were filtered using the following parameters: (i) proteins identified with high false discovery rates (FDRs) and with more than one peptide, (ii) proteins with a *p*-value of 0.05 (or less), and (iii) proteins with a fold change of 2. (**A**) Pie chart showing the number and percentage of up- and downregulated proteins. (**B**) Number and percentage of proteins from Figure 5A containing KFERQ-like motifs as analyzed using https://rshine.einsteinmed.org (accessed on 9 February 2022). (**C**) The number and percentage of proteins containing KFERQ-like motifs that are up- and downregulated in PA and SA. (**D**) The number and percentage of proteins containing different KFERQ-like motifs.

Having determined that CMA activity decreases in POMC-like cells exposed to PA and SA, we asked about the putative proteins whose degradation through CMA could be affected by the presence of PA and SA in the hypothalamic cells. In addition to the identification of such proteins, we asked which cellular processes could be affected by the presence of these SatFAs. To carry out this analysis, lysosomal fractions were isolated from POMC-like cells (as described in Figure 2A) previously incubated with heavy amino acids (Lys6, Arg6) and treated for 6 h with BSA, PA, and SA. Once each lysosomal fraction was obtained, the proteins in these fractions were identified through SILAC-based proteomics. Data analysis was performed by Mascot 2.5.1 coupled to Proteome Discoverer 2.2 and the Swissport database. The data were analyzed as the proteins present in PA with respect to BSA (named PA) or SA with respect to BSA (named SA). The results identified 1833 proteins in the PA and 1969 proteins in the SA lysosomal samples. Then, the data were filtered using the following parameters: (i) proteins identified with high false discovery rates (FDRs) and with more than 1 peptide, (ii) proteins with a *p*-value ≤ 0.05, and (iii) proteins with a fold change of ±2. Considering these parameters, 152 and 97 proteins in the PA and SA samples, respectively, were identified (Supplementary Table S1). Of these proteins, around 43% and 48% were upregulated in PA and SA, whereas 57% and 52% were downregulated in the PA and SA lysosomal samples, respectively (Figure 5A). To identify the proteins that were putatively degraded by CMA, the data (Supplementary Table S1) were analyzed with the online tool https://rshine.einsteinmed.org (accessed on 9 February 2022), which identifies canonical and non-canonical KFERQ-like motifs. In the PA and SA lysosomal samples, more than 85% of the proteins were found to contain KFERQ-like motifs (Figure 5B and Supplementary Table S2). Of these proteins, also in both samples, around 47% were found to be upregulated and 53% downregulated (Figure 5C and Supplementary Table S2). Regarding the type of KFERQ-like motif, in the PA and SA lysosomal samples identified, 22% of proteins were identified as having canonical motifs, 21–24% with phospho-activated motifs, 25% with acetyl-activated motifs, 17% with phospho- and acetyl-activated motifs, and 12–13% with the 3 types of motifs (Figure 5D and Supplementary Table S2). 

**Figure 6:**
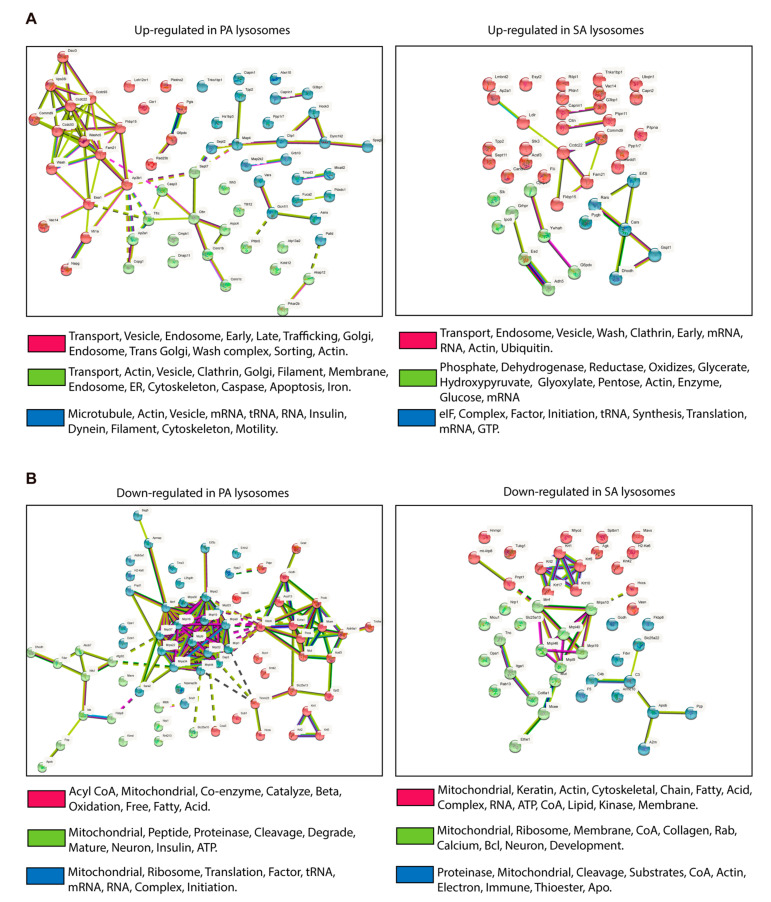
CMA-dependent pathways putatively affected in POMC-like neurons treated with PA and SA. The KFERQ-like motifs containing proteins (Figure 5B) that were found to be up- and downregulated were introduced in STRING (https://string-db.org, accessed on 9 February 2022) to analyze the cellular pathways in which they interacted in. Three interacting clusters were chosen (shown in red, green, and blue). (**A**) STRING outputs showing the principal pathways in which the upregulated proteins in PA and SA participated in. (**B**) STRING outputs showing the principal pathways in which the downregulated proteins in PA and SA participated in.

The proteins containing KFERQ-like motifs (Supplementary Table S2) were analyzed by STRING (https://string-db.org, accessed on 9 February 2022) to identify the cellular pathways in which they participated in. Among others, the proteins that were putatively more degraded (upregulated in the lysosomal samples) belong to vesicle transport (mainly endosomes), ER and Golgi trafficking, and cytoskeleton remodeling proteins (Figure 6A). However, the principal proteins that were putatively less degraded through CMA with the PA and SA treatment (downregulated in the lysosomal samples) belong to mitochondrial processes, such as translation and β-oxidation (Figure 6B). Overall, these analyses identified, in lysosomal samples, proteins whose putative degradation through CMA can be altered in POMC neurons exposed to PA and SA. 

### 3.4. PA and SA Can Affect the Insulin Response in POMC-like Neurons by Inhibiting CMA Activity

**Figure 7:**
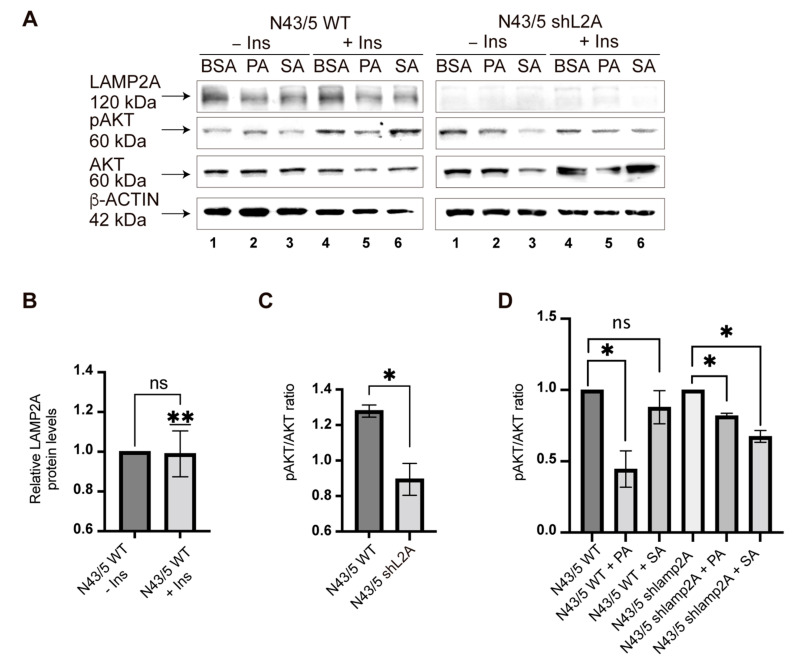
PA and SA can affect the insulin signaling response in POMC-like neurons by inhibiting CMA. (**A**) N43/5 wild-type and N43/5 shL2A C2 cell lines were treated with BSA or 100 μM of PA or SA for 6 h and then stimulated with insulin. The insulin response was evaluated by Western blot using pAKT antibody. Total AKT and β-ACTIN antibodies were used as a control. (**B**) The graph compares the basal (BSA) LAMP2A protein levels in N43/5 WT with or without insulin stimulation (lines 1 and 4). The quantification was analyzed using an unpaired *t* test and a one sample *t* test (*n* = 3). (**C**) The graph compares the basal (BSA) pAKT/AKT ratios between WT and N43/5 shL2A cells with or without insulin stimulation (lines 1 and 4). The quantification was analyzed using an unpaired *t* test (*n* = 3). (**D**) The basal (BSA) pAKT/AKT ratios (Figure 7B) were considered as 1 and compared with the pAKT/AKT ratios of the cells pretreated with PA and SA (ratio lines 1 and 4 versus, ratio lines 2 and 5, or lines 3 and 6). The quantification was analyzed using an unpaired *t* test (*n* = 3). Note: before calculating the pAKT/AKT ratios, every pAKT and AKT signal was normalized against its respective β-ACTIN signal. n.s. = non-significant, * = *p* < 0.05, ** = *p* < 0.01.

Interestingly, the previous analyses showed that insulin-degrading enzyme (IDE) and growth factor receptor-bound protein 10 (Grb10), two proteins involved in the insulin signaling pathway [34,35,36,37,38], contain KFERQ-like motifs (Supplementary Table S2). However, nothing is known about whether CMA activity is important for insulin signaling in POMC neurons. To evaluate this issue, we first developed a cell line with reduced LAMP2A protein levels through lentiviral transduction of an shRNA against *lamp2a* (Supplementary Figure S1). After the infection, the expression of LAMP2A protein was evaluated by Western blot in two clones (N43/5 shL2A C1 and N43/5 shL2A C2). We observed 75% less LAMP2A protein in the clone N43/5 shLA2A C2 than in the control; thus, we used this clone to evaluate the insulin response (Supplementary Figure S1). We treated the N43/5 wild-type cells (WT) or the N43/5 shL2A C2 (N43/5 shL2A) with BSA or 100 μM of PA and SA for 6 h and then stimulated the cells with insulin (Figure 7A). First, we observed that insulin treatment does not affect the LAMP2A protein levels in the N43/5 WT cell line under basal conditions (BSA) (Figure 7A,B). Next, the insulin response was evaluated by assessing the AKT phosphorylation (pAKT) by Western blot. Under basal conditions, we found that the response to insulin stimulation was reduced by around 30% in the N43/5 shL2A cells compared with the WT cells (Figure 7A,C). As previously reported [23], we observed that the presence of PA inhibited around 45% of the insulin response of the WT cells (Figure 7A,D). However, compared with BSA, the non-significant results suggested that the incubation with SA inhibited around 15% of the insulin response in WT cells (Figure 7A,D). In N43/5 shL2A cells, and compared with BSA, the treatment with both PA and SA inhibited the insulin response by around 20% and 30%, respectively (Figure 7A,D). Overall, these results support the fact that, in addition to macroautophagy, CMA activity also contributes to the regulation of the insulin response in POMC neurons. Further, principally, PA could affect the insulin response by impairing CMA activity in POMC neurons.

## 4. Discussion

Obesity is a worldwide pandemic problem that increases the risk of developing cardiovascular diseases, non-alcoholic fatty liver, and insulin resistance (diabetes mellitus type 2), among others [2,39]. The hypothalamus can directly impact on obesity development as it is a tissue that regulates food intake by sensing the circulating levels of glucose and glucagon [3]. Among other cells, the hypothalamus’ function is carried out by proopiomelanocortin (POMC) neurons that inhibit the appetite [3]. Thus, dysregulation of POMC neurons could induce or favor an obesogenic condition. One of the principal factors inducing an obesity condition is the consumption of unhealthy foods, such as high-fat diets (HFDs) containing high levels of saturated fatty acids (SatFAs), such as palmitic (PA) and stearic (SA) acids [40]. Studies have shown that SatFAs, especially PA, accumulate in the hypothalamus in rodents fed an HFD [4] and that one of the consequences of SatFAs’ accumulation in the hypothalamus is the inhibition of macrouatophagy in POMC neurons, which, consequently, affect the insulin response of these neurons [22,23]. 

In this study, we showed in vitro that PA and SA reduce the total and lysosomal protein levels of LAMP2A (Figure 1 and Figure 2) and inhibit chaperone-mediated autophagy (CMA) activity in hypothalamic-like POMC neurons (Figure 3 and Figure 4). By isolating the lysosomal fractions of cells treated with PA and SA, we found that 85% of the proteins in this fraction have KFERQ-like motifs and, from here, that around 47% were upregulated (53% downregulated) after treatment with PA or SA (Figure 5). Using STRING analysis, we determined that the up- and downregulated CMA putative substrates belong, principally, to vesicle formation and trafficking and to mitochondrial processes, such as translation and β-oxidation (Figure 6). Lastly, our data show that CMA inhibition can affect the insulin response of POMC-like neurons (Figure 7). 

Thus, our work indicates for the first time that PA and SA inhibit the activity of CMA in POMC-like neurons in vitro. Such inhibition could be a consequence of reduced total and lysosomal LAMP2A protein levels in cells exposed to PA and SA. This result is in line with other reports showing that the levels of LAMP2A change in a hypothalamic cell line treated with PA [20]. Regarding the mechanisms regulating LAMP2A protein levels, it has been shown that LAMP2A degradation is performed through CMA-specialized lysosomes [41]. More specifically, it was reported that lysosomes isolated from the hepatocytes of mice fed an HFD show greater LAMP2A degradation due to changes in the lipid content of the lysosomal membrane [26]. Thus, PA and SA could alter the lysosomal membrane and induce an increase in LAMP2A degradation through CMA-specialized lysosomes; however, this remains to be confirmed. On the other hand, although the latter could explain the reduction that we observed in lysosomal LAMP2A after PA and SA treatments, the exact mechanism by which PA and SA reduce the total LAMP2A protein levels remains to be completely investigated. 

Based on the presence of KFERQ-like motifs, different studies have reported proteomics analyses of putative CMA substrates under different conditions. The results of these analyses have demonstrated that (i) CMA could drive the degradation of around 75% of the human proteome [16], (ii) that CMA activity is crucial to preventing the collapse of neuronal proteins prone to aggregation [42], and (iii) to understand that there are KFEQR-like motif-containing proteins whose abundance is dependent or independent of the CMA activity in SUM159 cancer cells [43]. In this sense, our work contributes to identifying the abundance and nature of KFERQ-like motif-containing proteins present in the lysosomes of POMC neurons exposed to PA and SA. Interestingly, up- and down-regulated proteins participate in different cellular pathways, involved in vesicle formation and trafficking (upregulated) and mitochondrial processes (downregulated). However, it remains to be elucidated whether the observed changes occur in vivo and whether they correspond to a physiological adaptation of the cell metabolism towards the presence of PA and SA or to a pathological effect of the SatFAs (CMA activity alteration). 

Among the autophagy pathways, macroatophagy is the only one that has been involved in crosstalk with the insulin response. Different reports have shown that a deficiency in macroautophagy induces insulin resistance in liver cells and impairs insulin secretion in the pancreas [24,25]. In the context of the hypothalamus, the specific modulation of macroautophagy and autophagy-related genes in POMC neurons modulates obesity and conduces metabolic disorders and insulin resistance [23,44]. Our results show that the downregulation of LAMP2A protein levels affects the insulin response of POMC-like neurons, suggesting that in addition to macroautophagy, CMA activity also participates in hypothalamic insulin signaling. The exact mechanism by which CMA can regulate insulin signaling in POMC remains to be clarified. However, it can be hypothesized that CMA could drive the degradation of protein involved in the insulin response. In this sense, we found that proteins participating in the insulin signaling cascade, such as Grb10 [36,37,38] and IDE [34,35], contain KFERQ-like motifs and were observed to be up- and downregulated in PA lysosomal samples, respectively. Despite this, additional experiments need to be performed to confirm whether IDE and Grb10 are CMA substrates and whether they have a role in insulin signaling in POMC under PA and SA exposure.

Lastly, in agreement with other reports, we showed that PA (and to a lesser extent SA) inhibits the insulin response of POMC-like neurons [23]. Interestingly, the inhibiting effect of PA on insulin response was reduced in cells with low LAMP2A protein levels, suggesting that PA can affect the insulin response, in part, by inhibiting CMA activity. 

Overall, our in vitro data indicate that PA and SA can inhibit CMA activity in POMC-like neurons and could impact pathways related to vesicle transport, mitochondrial function, and insulin response. Although this study must be validated in vivo, it suggests that CMA can be considered as a new target of the pathological effects of HFDs rich in SatFAs on the hypothalamus.

## Data Availability

The data presented in this study are available on request from the corresponding authors.

## Supplementary Materials

The following supporting information can be downloaded at https://www.mdpi.com/article/10.3390/cells11060920/s1. Figure S1: Generation of a stable N43/5 cell line with reduced LAMP2A protein levels, Table S1: up- or downregulated proteins in lysosomal samples upon PA and SA treatment, Table S2: KFERQ-motifs analysis of proteins changing in lysosomal samples upon PA and SA treatment.
