# Supplementary material for: Palmitic and Stearic Acids Inhibit Chaperone-Mediated Autophagy (CMA) in POMC-like Neurons In Vitro
Source: Cells. 2022 Mar 8;11(6):920. doi: 10.3390/cells11060920 (PMC8945987; doi:10.3390/cells11060920)
Supplement: Supplementary file 1 [file cells-11-00920-s001.zip › Supplementary data/Supplementary Figure S1.pdf]

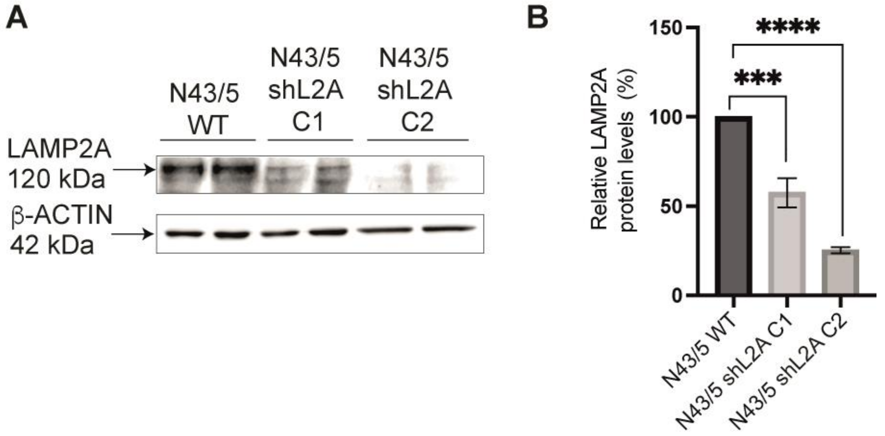

Supplementary Figure S1: Generation of a stable N43/5 cell line with reduced LAMP2A protein levels. (A) Stably cell line POMC like neurons with reduced LAMP2A protein levels were generated through lentiviral infection encoding for a shRNA against *lamp2a* (N43/5 shL2A). LAMP2A protein levels of two different clones (N43/5 shL2A C1 and C2) were evaluated by western blot using an anti-LAMP2A antibody. β–ACTIN was evaluated as loading control. (B) quantification from Supplementary Figure S1A using ordinary one-way ANOVA (n=4). * *= p < 0.01, *** = p < 0.001.
